# Dual inhibition of P38 MAPK and JNK pathways preserves stemness markers and alleviates premature activation of muscle stem cells during isolation

**DOI:** 10.1186/s13287-024-03795-0

**Published:** 2024-06-21

**Authors:** Teoman Ozturk, Julien Mignot, Francesca Gattazzo, Marianne Gervais, Frédéric Relaix, Hélène Rouard, Nathalie Didier

**Affiliations:** 1 Univ Paris Est Creteil, INSERM, EFS, IMRB, 94010 Creteil, France; 2 EnvA, IMRB, 94700 Maisons-Alfort, France; 3 AP-HP, Hopital Mondor, Service d’histologie, 94010 Creteil, France

**Keywords:** Muscle stem cells, PAX7, MAPK signaling pathways, Enzymatic dissociation, Cell therapy

## Abstract

**Background:**

Adult skeletal muscle contains resident muscle stem cells (MuSC) with high myogenic and engraftment potentials, making them suitable for cell therapy and regenerative medicine approaches. However, purification process of MuSC remains a major hurdle to their use in the clinic. Indeed, muscle tissue enzymatic dissociation triggers a massive activation of stress signaling pathways, among which P38 and JNK MAPK, associated with a premature loss of MuSC quiescence. While the role of these pathways in the myogenic progression of MuSC is well established, the extent to which their dissociation-induced activation affects the functionality of these cells remains unexplored.

**Methods:**

We assessed the effect of P38 and JNK MAPK induction on stemness marker expression and MuSC activation state during isolation by pharmacological approaches. MuSC functionality was evaluated by in vitro assays and in vivo transplantation experiments. We performed a comparative analysis of the transcriptome of human MuSC purified with pharmacological inhibitors of P38 and JNK MAPK (SB202190 and SP600125, respectively) *versus* available RNAseq resources.

**Results:**

We monitored PAX7 protein levels in murine MuSC during muscle dissociation and demonstrated a two-step decline partly dependent on P38 and JNK MAPK activities. We showed that simultaneous inhibition of these pathways throughout the MuSC isolation process preserves the expression of stemness markers and limits their premature activation, leading to improved survival and amplification in vitro as well as increased engraftment in vivo. Through a comparative RNAseq analysis of freshly isolated human MuSC, we provide evidence that our findings in murine MuSC could be relevant to human MuSC. Based on these findings, we implemented a purification strategy, significantly improving the recovery yields of human MuSC.

**Conclusion:**

Our study highlights the pharmacological limitation of P38 and JNK MAPK activities as a suitable strategy to qualitatively and quantitatively ameliorate human MuSC purification process, which could be of great interest for cell-based therapies.

**Supplementary Information:**

The online version contains supplementary material available at 10.1186/s13287-024-03795-0.

## Background

Adult skeletal muscle has a remarkable regenerative capacity sustained by muscle stem cells (MuSC) also called satellite cells. This resident population of MuSC is anchored within a complex microenvironment (the niche) between the basal lamina and the sarcolemma of the myofibers which guarantees the maintenance of their quiescent state in homeostatic conditions, and closely regulates their behavior during regeneration [1, 2]. Upon injury or stress stimuli, quiescent MuSC can activate and divide, to subsequently differentiate and fuse to form new myofibers, or self-renew to restore the pool of quiescent MuSC [3, 4]. Quiescent MuSC express PAX7, a key transcription factor for the preservation of their stemness and engraftment potential upon transplantation in vivo [5, 6].

By virtue of their high myogenic potential and their ability to self-renew, MuSC are an enticing source of cells for clinical applications [7]. Indeed, freshly isolated MuSC transplanted in regenerating muscle exhibit potent engraftment potential [8, 9]. However, their clinical use is still hampered by their limited number in adult muscle declining with aging [10], as well as their replicative senescence and the loss of their therapeutic potential during in vitro expansion [11, 12]. In addition, the isolation process of pure population of MuSC is time-consuming, tedious, and often result in suboptimal purification yields [13–15]. Indeed, the thorough enzymatic dissociation of the muscle tissue required to extract MuSC from their native niche, induces immediate quiescence break initiated by a Rac1 to Rho GTPase switch [16]. Moreover, a massive activation of stress pathways was reported in cells from digested tissues, including skeletal muscle tissue [17, 18]. Accordingly, rapid and significant transcriptional changes were observed in MuSC during muscle dissociation leading to a redefinition of the quiescent MuSC transcriptome [19, 20]. Transcriptional events induced by muscle dissociation in MuSC mirror those occurring following acute muscle damage [17, 21]. As soon as 15 min of dissociation, immediate and early response genes and members of the AP-1 family genes are up-regulated, consistent with the rapid activation of the MAPK pathways, ERK, P38 and JNK. Next, expression of genes coding for quiescence markers and Notch signaling targets progressively declines, while activation associated-genes are up-regulated [17]. Among the MAPK family, P38 and JNK are both activated in response to environmental stress signals. It is now well established that the P38 MAPK signaling pathway plays a pivotal role in early activation of MuSC in stress conditions, in their progression in the myogenic program as well as their self-renewal in vitro [22–28]. Conversely, the role of JNK in MuSC behavior regulation has been less extensively studied. However, the small GTPases Rac and Cdc42 were previously shown to activate JNK in fibroblasts [29], and increased level of activated JNK was reported in freshly isolated MuSC [30], supporting that JNK could be an early player in dissociation-induced MuSC activation. Activated JNK translocates to the nucleus where it regulates the activity of multiple transcription factors including cJUN. Phosphorylated cJUN, together with FOS or ATF proteins, form a dimeric complex named AP-1 (Activating Protein-1) [31]. In the context of dissociation, these transcription factors are described as some of the earliest known transcriptional effectors involved in MuSC early activation [17, 21, 32].

While it is obvious that MAPK pathways are key players in MuSC activation and myogenic progression, the impact of their massive activation in response to muscle dissociation on MuSC functionality is still unclear. Using pharmacological tools, we explored the role of P38 MAPK and JNK on dissociation-induced early activation of MuSC. We monitored PAX7 protein levels during muscle dissociation, demonstrating a two-step reduction. We showed that combined inhibition of these pathways during the whole isolation process preserves stemness marker expression and limits the early activation of MuSC, improving their amplification rate in vitro and their engraftment potential in vivo. Overall, our data demonstrate that restricting the massive activation of MAPK signaling pathways from the beginning has a beneficial effect on MuSC functionality later on and could therefore be a suitable approach for cell therapy strategies.

## Methods

This study was carried out and reported in line with ARRIVE guidelines 2.0.

### Enzymatic dissociation of skeletal muscles

Mice were euthanized by cervical dislocation and the death was confirmed by cardiorespiratory arrest. TA muscles were harvested in HBSS added with 0.2% BSA and 0.5% antibiotics (HBSS/BSA), vehicle (DMSO) or Pharmacological Inhibitors 0.1X (noted ± PI). 1X concentrations of PI were 10 µM for SB202190 (Sigma) and 2.5 µM for SP600125 (Sigma). Single TA muscles were mechanically minced with scissors in Digestion Solution (DS), consisting in Collagenase A (2 mg/ml, Roche), Dispase II (3 mg/mL, Roche), DNase I (10 µg/mL, Roche), 0.4 mM CaCl_2_, 5 mM MgCl_2_, ± PI 1X. TA muscles were digested for 40 min at 37 °C, with gently shaking every 10 min. After 20 min, the muscle lysate was left to decant, and 1 mL of the upper phase was recovered in HBSS/BSA ± PI 1X and kept on ice. In parallel, 1 mL of fresh DS was added to the digestion tube and the digestion was proceeded for additional 20 min. After dissociation, the cell suspension was filtered through a 40-µm nylon filter and centrifuged at 300 g for 10 min. Cells were incubated with Red Blood Cell lysing buffer ± PI 1X for 10 min, washed with HBSS ± PI 0.1X and transferred in a flow cytometry collection tube for immunostaining. The processing order of the different conditions was changed between independent experiments.

### Enzymatic dissociation of single isolated fixed TA muscles

TA muscles were directly fixed upon harvest with cold 0.5% PFA for 10 min and then minced. Cells were left in cold PFA for 1 h, washed with HBSS and then dissociated following the same protocol mentioned above.

### MuSC immunostaining for flow cytometry analysis

Mononucleated cells from dissociated TA muscle were incubated with Fixable Viability Stain 780 (BD Biosciences) ± PI 1X for 10 min. Cells were washed with HBSS ± PI 0.1X, and then fixed and permeabilized using Transcription Factor buffer set (BD Pharmingen), according to manufacture instructions. Fixed cells were incubated with blocking solution containing 5% BSA/1% Mouse Serum, for 15 min and then with 20 ng of coupled antibodies for PAX7 and MYOG and with 80 ng of coupled antibodies for p-HSP27, p-cJUN and MYOD (Table S2), for 50 min at room temperature. Cells were washed and resuspended in Permeabilization/Wash buffer. Analyses were performed using a BD FACS Canto™ Flow Cytometer (BD Biosciences).

### MuSC isolation by fluorescence activated cell sorting

Mononucleated cells from digested muscles were incubated for 45 min on ice with coupled antibodies (Tables S3 and S4). Vehicle or PI 1X were added during the whole process.

### Cell culture

Purified MuSC were plated at clonal density (400 cells/ cm^2^) on gelatin-coated flasks or 8 wells µ-slides (Sarstedt) in DMEM (Gibco) added with 20% Fetal Bovine Serum (Gibco), 10% horse serum (Life Technologies), 5 ng/mL basic FGF (Prepotech), 0.5% antibiotics, and kept in culture for 4 days. Fresh medium was added after 2 days of culture, and dead cells in the removed medium were counted using Trypan Blue and a Hemocytometer.

### Genetically modified mouse models

*Tg:Pax7-nGFP* transgenic mice express a nuclear enhanced Green Fluorescent Protein under the control of the murine *Pax7* promoter [33]. As PAX7 is a specific marker of MuSC in adult muscle, this reporter line is commonly used to identify these cells in flow cytometry experiments. In addition, the stability of the nGFP protein enables to monitor the progression of these cells in the myogenic program (i.e. PAX7 + cells and their progenies). *Rosa*^*nT−nG*^ mice harbor a nuclear-targeted two-color fluorescent Cre reporter allele composed of a *loxP*-flanked *nT* cassette and a *nG* cassette, under the control of a *CMV* promoter, inserted into the *ROSA26* locus [34]. In absence of Cre-mediated recombination, cells derived from these mice express a nuclear-localized Tomato fluorescence whereas GFP is not expressed. *Pax7*^*creERT2*^ mouse line was generated by inserting an *iresCreERT2* cassette, downstream of the stop codon of the *Pax7* gene [35]. *Rosa26iDTR-loxP* mice carry a *loxP*-flanked STOP cassette upstream of the open reading frame of the simian Diphtheria toxin receptor (*DTR*), inserted into the *ROSA26* locus [36]. Cells expressing this Cre-inducible DTR (iDTR) are ablated following Diphtheria toxin administration. *Rag2*^*−/−*^*γC*^*−/−*^ mice exhibit T cell, B cell and NK cells immunodeficiencies due to *Rag2* and *Il2rg* gene invalidation, making them suitable hosts for cell transplant experiments [37].

### MuSC transplantation

For these experiments, recipient *Pax7*^*creERT2*^*;iDTR-loxP;Rag2*^*−/−*^*γC*^*−/−*^ mice were generated by crossing *Pax7*^*creERT2*^*, Rosa26iDTR-loxP* and *Rag2*^*−/−*^*γC*^*−/−*^ mouse lines mentioned above [35–37]. This immunodeficient mouse model enables partial depletion of endogenous MuSC pool, thereby improving the engraftment efficiency of transplanted cells [38, 39]. Recipient mice received intraperitoneal injections of Tamoxifen (150 µg/g of mouse) for 4 consecutive days. Tamoxifen induces nuclear translocation of the Cre recombinase expressed specifically in PAX7 + MuSC. Cre-mediated recombination leads to the excision of the STOP cassette located upstream of the *DTR* transgene, thus enabling DTR expression specifically in MuSC. The following day, TA muscle of recipient mice was injured by intramuscular injection of Cardiotoxin (10 μM). Three days later, Diphtheria toxin (1 ng/g total body weight, Sigma) was injected intramuscularly in a small volume (15 µL) to deplete endogenous DTR expressing MuSC. Eight hours later, 5.10^4^ MuSC purified from reporter mice were injected in the injured TA using a Hamilton syringe [31]. After 2 weeks, TA muscles grafted with *Tg:Pax7-nGFP* MuSC were dissociated with SB + SP, following the protocol above. Cells were transferred in a Trucount Tube (BD Biosciences). Acquisitions were performed on 20 000 beads and total number of GFP cells was determined according to the total number of beads. One month after cell injection, TA muscles grafted with *Rosa*^*nT−nG*^ MuSC were fixed in 4% PFA for 24 h, transferred in 20% sucrose solution overnight and then snap frozen in liquid nitrogen-cooled isopentane. Serial muscle sections (10 µm) were fixed for 15 min with 1.5% PFA, permeabilized 10 min with 0.2% Triton X-100, blocked 60 min with 5% BSA and 30 min with anti-mouse IgG Fab fragment (Jackson Laboratories). Sections were incubated with the primary antibodies overnight, and then with coupled secondary antibodies (Table S5) and mounted with Prolong Diamond (ThermoFisher). For these surgical procedures, mice were anesthetized by inhalation of isoflurane (4%) maintained at 3% throughout the procedure and euthanized by cervical dislocation.

### RT-qPCR on freshly isolated MuSC

MuSC were directly sorted in lysis buffer from RNA extraction Kit (#74034, Qiagen) and RNA were purified according to manufacturer recommendations. Reverse-transcription was performed using the Superscript IV Reverse Transcriptase Kit (ThermoFisher) with random primers, and qPCR using the Power SYBR Green PCR Master Mix (Sigma). See the list of the primers in Table S6.

### RNA-seq on human MuSC

hMuSC were directly sorted in the RNA extraction Kit lysis buffer. cDNA libraries were generated with 1 ng of total RNA using the Illumina Stranded total RNA prep Ligation Kit (See also Supplemental experimental procedure).

### Image analysis

For both optical and immunofluorescence analysis, images were acquired either with a Zeiss LSM800 confocal microscope or a Zeiss D1 Observer.

### Statistic and reproducibility

Results are represented as the mean ± SEM. Statistical analysis were performed using GraphPad Prism software. Statistical significance was determined by One and Two-Way ANOVA tests followed by Tukey’s or Dunnett’s multiple comparisons tests. *p* < *0.05* was considered significant (*p < 0.05, **p < 0.01, ***p < 0.001, ****p < *0.0001*). ns represents statistically not significant.

## Results

### Muscle dissociation induces a marked decline in PAX7 protein

**Fig. 1:**
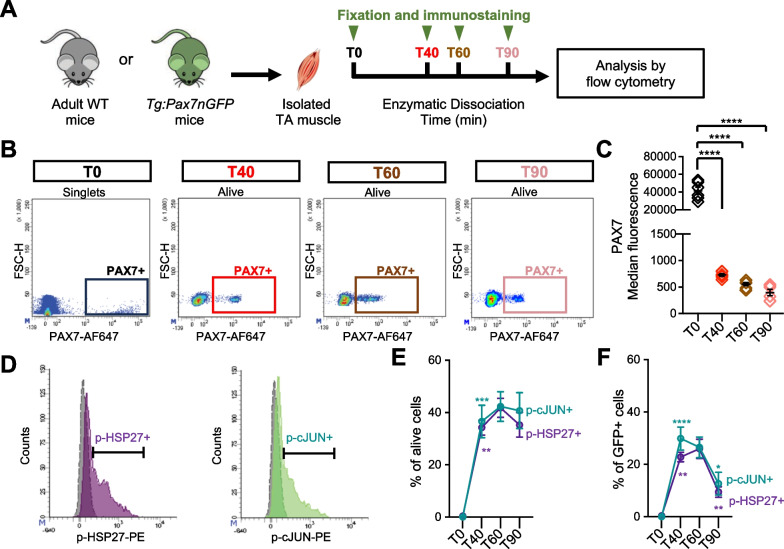
PAX7 protein declines drastically in MuSC concomitant with activation of stress signaling pathways upon dissociation. **A** Isolated TA muscles were either fixed directly after harvest (T0) or dissociated for different times (T40, T60 and T90) before fixation and immunostaining. **B** Representative density scatter plots showing PAX7 expression after the different times of dissociation. Debris, doublets and dead cells were excluded from the analysis, except for T0 when muscles were directly fixed. **C** Median fluorescence of PAX7 immunostaining analyzed by flow cytometry (n = 6 mice for each time point). **D** Representative histograms showing phospho-HSP27 (p-HSP27, purple) and phospho-c-JUN (p-cJUN, green) cells among alive cells at T60. Positivity was determined based on FMO (grey). **E**, **F** Proportions of alive cells (**E**) and GFP + cells (**F**), with detectable levels of p-HSP27 or p-cJUN in TA muscles from *Tg:Pax7nGFP* mice, determined by flow cytometry (n = 8 mice for T40, T60 and T90, n = 3 mice for T0). Values are the mean ± SEM of independent experiments. One- and Two-Way ANOVA with Tukey’s multiple comparisons test, *p < 0.05, **p < 0.01, ***p < *0.001* and ****p < *0.0001* (*versus* preceding time point for E and F)

Recent studies have highlighted profound transcriptomic changes in MuSC during muscle tissue dissociation, notably a reduction in *Pax7* transcripts [19]. We therefore sought to investigate the dynamics of PAX7 regulation at the protein level during this process, by flow cytometry (Fig. 1). The level of PAX7 protein in quiescent MuSC was determined by analyzing mononucleated cells from single Tibialis Anterior (TA) muscle fixed directly upon harvest (T0). For other timepoints, mononucleated cells were recovered after 40, 60 and 90 min of dissociation (T40, T60 and T90), and immediately fixed and immunostained for PAX7 as previously described [40] (Fig. 1A). Between T0 and T40, we noted a strong reduction in PAX7 protein levels in the entire population of MuSC, evidenced by the significant reduction in the median of fluorescence of PAX7 staining (Figs. 1B, C and S1A, B). Subsequently, from T40 to T90, the medians of fluorescence continue to decrease, albeit in a much less pronounced manner. In addition, at T40, PAX7 + population was well defined and grouped, showing a homogenous level of PAX7 protein. Conversely, from T60 onwards, the PAX7 + population appeared more dispersed, demonstrating a progressive loss of PAX7 protein but at different rates among the MuSC population (Fig. 1B). Overall, our observations revealed that PAX7 proteostasis is rapidly affected upon dissociation leading to a two-step decrease. A rapid and strong decrease occurring in the entire population of PAX7 + MuSC, followed by a more heterogeneous and progressive decline.

Since P38 MAPK and JNK pathways are prominently induced pathways during the dissociation process [17, 30], we next evaluated whether they could be involved in the downregulation of PAX7 protein. We first investigated by flow cytometry the activation kinetics of both pathways by monitoring the phosphorylation status of HSP27 and c-JUN (referred to as p-HSP27 and p-cJUN), known downstream targets of P38 MAPK and JNK, respectively. For these analyses, we used *Tg:Pax7nGFP* mice, which enabled us to follow the myogenic lineage even if PAX7 expression was lost through perdurance of the GFP protein [33]. We assessed the proportion of p-HSP27 + and p-cJUN + cells, among the whole population of mononucleated cells (alive cells), and the GFP + myogenic cells (Figs. 1D–F and S1C–F). At T0, almost all the cells were negative for p-HSP27 and p-cJUN consistent with the homeostatic state of cells in adult muscle. The proportions of mononucleated cells p-HSP27 + and p-cJUN + significantly increased at T40 compared to T0, continued to increase until T60, and finally began to decrease at T90. Activation of P38 MAPK and JNK pathways is therefore coordinated and peaks at 60 min of dissociation in muscle mononucleated cells (Fig. 1E). A concurrent activation of these two pathways was also observed in GFP + myogenic cells between T0 and T40 (Fig. 1F). However, in contrast to mononucleated cells, the levels of GFP + p-HSP27 + or p-cJUN + cells, were significantly diminished at T90 compared to T60. Overall, these experiments demonstrated that P38 and JNK pathway activation is a more transitory event in MuSC than in other mononucleated cells of the muscle (Fig. 1E, F).

**Fig. 2:**
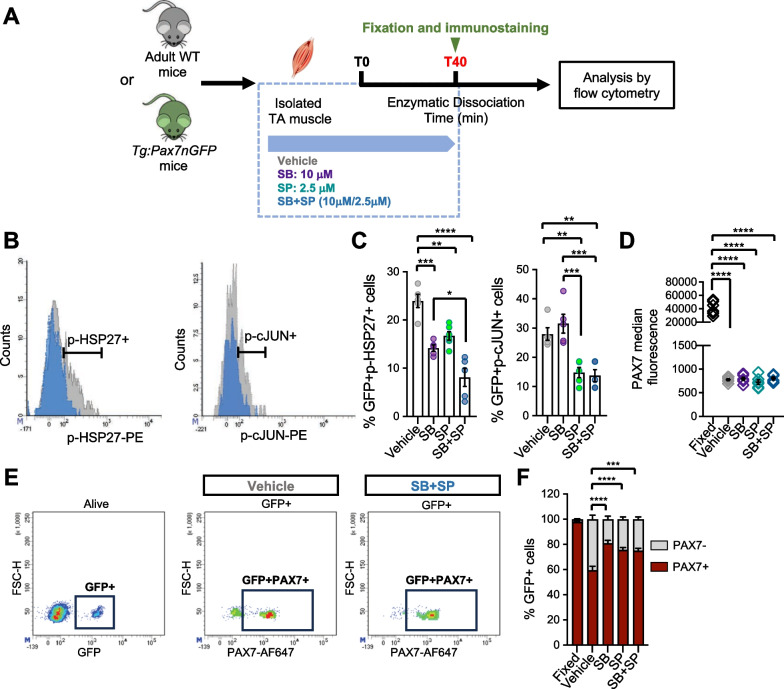
Inhibition of P38 MAPK and JNK pathways does not prevent initial drop of PAX7 protein. **A** Inhibitors of P38 MAPK (SB202190, SB) and JNK (SP600125, SP) or vehicle were added from the muscle harvest to the fixation. *Tg:Pax7nGFP* mice were used to monitor GFP + myogenic cells and WT mice to monitor PAX7 levels. **B** Representative histograms showing phospho-HSP27 (p-HSP27) and phospho-cJUN (p-cJUN) positive cells among the GFP + population from TA digested with vehicle (grey) or SB + SP (blue). **C** Proportion of GFP + p-HSP27 + and GFP + p-cJUN + cells after 40 min of dissociation with vehicle or the inhibitors (n = 5 mice/group). **D** Median fluorescence of PAX7 immunostaining, determined by flow cytometry (n = 6 mice/group). **E** Representative density scatter plots showing the PAX7 + cells among GFP + population. **F** Proportion of PAX7 + cells among GFP + population (n = 4 to 5 mice/group). Debris, doublets and dead cells were excluded from the analysis. Values are the mean ± SEM of independent experiments. One Way and Two Way ANOVA and Tukey’s multiple comparisons test, *p < *0.05*, **p < 0.01, ***p < *0.001* and ****p < *0.0001*

Since P38 MAPK and JNK activation occurred concomitantly to the drastic decrease of PAX7 protein in MuSC, we next sought to determine whether these two phenomena were directly linked. We therefore added pharmacological inhibitors (PI) of these two pathways from the harvest of the muscle to the fixation step (Fig. 2). SB202190 (SB) and SP600125 (SP) were used alone or in combination as inhibitors of P38 MAPK and JNK, respectively (Fig. 2A). We first verified the efficacy of our treatments by assessing the proportion of GFP + p-HSP27 + and p-cJUN + cells in TA muscles from *Tg:Pax7nGFP* mice dissociated with vehicle, SB, SP or SB + SP for 40 min, by flow cytometry (Fig. 2B, C). We observed that in conditions with SB alone or with SB + SP, the proportion of GFP + p-HSP27 + cells was significantly reduced compared to vehicle. We also noticed a reduction in the proportion of GFP + p-HSP27 + with SP treatment that likely results from a direct effect of JNK on HSP27 phosphorylation state, as previously reported [﻿^41^, ^42^﻿]. In agreement, the combination of SB + SP had a synergistic inhibitory effect on the proportion of GFP + p-HSP27 + cells compared to SB treatment (Fig. 2C). Upon exposure with SP alone or in combination with SB, the proportion of GFP + p-cJUN + cells was reduced compared to control or SB conditions (Fig. 2C).

Having confirmed the inhibitory effect of our treatments, we next monitored the level of PAX7 protein in MuSC from TA muscles dissociated with vehicle, SB, SP or SB + SP (Fig. 2D). We observed that inhibition of P38 and/or JNK pathways did not prevent the drastic loss of PAX7 protein during the initial phase of muscle dissociation and that the mean level of PAX7 protein was similar between vehicle and treated conditions. Nevertheless, using the *Tg:Pax7nGFP* reporter line, we showed that treatment with inhibitors (alone or in combination) during the dissociation phase increased the proportion of GFP + cells exhibited detectable amounts of PAX7 (Fig. 2E, F). Our findings demonstrate that the sharp decline of PAX7 protein occurring in the initial phase of dissociation is independent of P38 MAPK and JNK increased activities. However, these pathways may rather contribute to the progressive and heterogenous loss of PAX7 observed later on.

### Inhibition of P38 MAPK and JNK throughout dissociation and cell sorting mitigates stemness markers down-regulation and early activation of MuSC

**Fig. 3:**
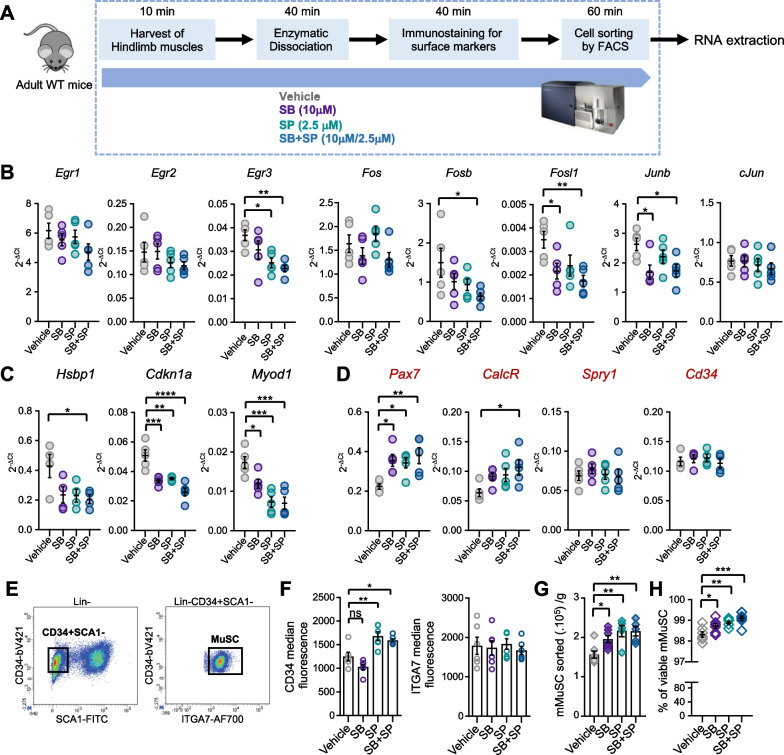
Lowering P38 and JNK activities preserves stemness marker expression and limits early activation of MuSC. **A** Hindlimb muscles from adult WT mice were digested for 40 min. Mononucleated cells were immunostained and MuSC purified by FACS (see Fig. S2). Vehicle or inhibitors were added during the whole process. Transcript levels of **B** *Egr* and AP-1 genes, **C** genes up-regulated upon activation, **D** genes coding for stemness markers, analyzed by RT-qPCR. Values are the mean ± SEM of 4 to 5 independent experiments. **E** Representative density scatterplots showing the expression of CD34 and SCA1 among the Lin- fraction, and of ITGA7 among CD34 + SCA1 + population. Graphs showing **F** the median level of fluorescence for CD34 and ITGA7 stainings, **G** the purification yield of murine MuSC (mMuSC) per g of muscle, **H** the percentage of viable mMuSC. Values are the mean ± SEM of 6 independent experiments (n = 6 mice/group). One-way ANOVA analysis and Dunnett’s multiple comparisons test, with *p < 0.05, **p < 0.01, ***p < *0.001* and ****p < *0.0001*

Since MuSC purification requires a cell sorting step after muscle dissociation, we next extended our inhibitory strategy to the entire process, including during immunostaining and FACS sorting (Fig. 3A). To uncouple the detrimental impact of prolonged enzymatic dissociation from the role of these pathways in early MuSC activation, we limited the dissociation time to 40 min. For these experiments, MuSC were recovered from hindlimb muscles of wildtype adult mice and purified as shown in Fig S2. We performed a transcriptomic profiling of MuSC purified from muscles digested and sorted in the presence of SB, SP, SB + SP or vehicle, by RT-qPCR. We first assessed the level of transcripts of immediate and early response genes (*Egr1*, *2*, *3*) and members of the AP-1 family genes (*cJun*, *Junb*, *Fos*, *Fosb* and *Fosl1*), previously shown to be strongly induced during MuSC isolation [17, 19, 20] (Fig. 3B). Among *Egr* genes, only *Egr3* transcripts were significantly downregulated in SP and SB + SP conditions compared to vehicle indicating that activated JNK was primarily involved in their increase during MuSC isolation (Fig. 3B). In agreement with previous observations, *Fos* transcript levels were stable regardless of the treatment [16]. *Fosb* and *Fosl1* transcripts exhibited a more pronounced decrease with SB + SP treatment, demonstrating the involvement of both JNK and P38 MAPK in their induction. *Junb* transcripts were similarly downregulated with SB and the combination SB + SP, proving that P38 MAPK was the main player. Overall, our data demonstrated that during dissociation, P38 MAPK and JNK contribute to the up-regulation of only a subset of immediate and early response genes.

We next analyzed the expression of markers described to be upregulated in early activated MuSC relative to quiescent MuSC, including *Myod1*, *Cdkn1a* (p21) and *Hspb1* (HSP27) (Fig. 3C). SB + SP treatment had the more marked effect on the transcript levels of these 3 genes, indicating that both P38 MAPK and JNK participate in their induction. Interestingly, the inhibition profiles obtained for *Myod1* transcripts suggested that JNK was predominantly involved. Overall, these results demonstrated that simultaneous inhibition of P38 MAPK and JNK during MuSC isolation restricts their premature activation. Of note, by flow cytometry analysis, we detected rare MYOD + cells in equivalent proportions under all digestion conditions, consistent with the timescale of purification (Fig. S2A, B). Moreover, SB + SP treatment synergistically increased the transcript levels of the stemness markers, *Pax7* and *Calcr*, while the levels of *Spry1* and *Cd34* transcripts remained unchanged (Fig. 3D). Interestingly, upon FACS sorting, we noticed an increased level of CD34 surface protein in SP and SB + SP conditions whereas of α7-integrin (ITGA7) level was similar (Figs. 3E, F and S2C–E). JNK induction during dissociation seems to affect CD34 protein level, which is essential for murine MuSC functionality and quiescent state [43]. This effect could rely on a post-translational regulation since a putative phosphorylation site by JNK was identified in the intracellular domain of CD34 [44].

Lastly, the purification yield of MuSC was significantly improved in all conditions with inhibitors, but more markedly with SP and SB + SP treatments (Fig. 3G). We hypothesize that this improved purification yields could be attributed to a better survival of the cells (Fig. 3H), associated with a better detection of CD34 + cells during the sorting step.

### Constant inhibition of P38 MAPK and JNK signaling pathways ameliorates survival and amplification rate of MuSC in vitro

**Fig. 4:**
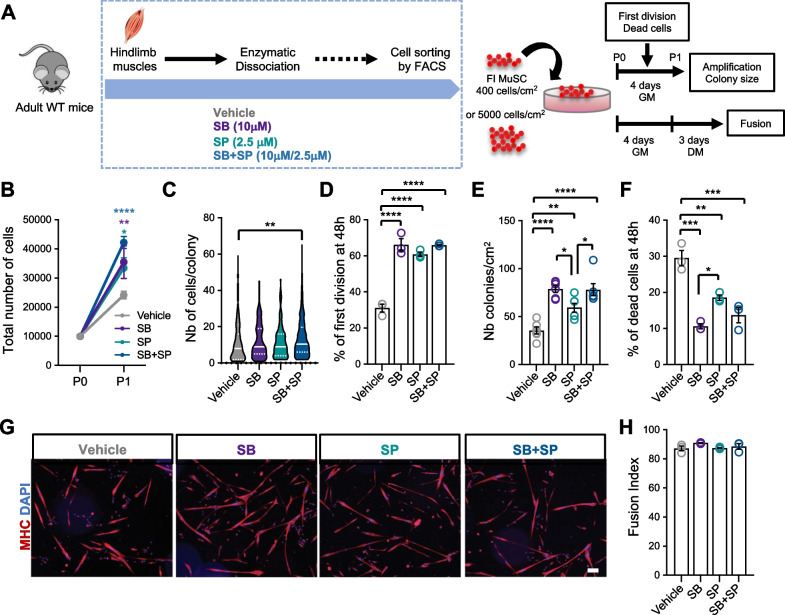
P38 and JNK inhibition during isolation elicits increased survival and amplification of MuSC in vitro*.* **A** MuSC were purified with vehicle or inhibitors and plated at low density in Growth Medium (GM) for 4 days or at high density for fusion (DM: Differentiation Medium). Graphs showing, **B** the total number of cells after 4 days of amplification, **C** the size of the colonies, **D** the proportion of cells having proceeded to their first division at 48 h, **E** the total number of colonies per cm^2^, **F** the proportion of dead cells relative to seeded cells 48 h after plating. **G** Representative images of the myotubes obtained after 3 days in DM, immunostained for Myosin Heavy Chain (MHC). Nuclei were stained with DAPI. Scale bar: 100 µm. **H** Fusion Index (%). Values are the mean ± SEM of minimum 3 independent experiments (n = 3–6 mice/group). One-way ANOVA analysis, with *p < *0.05*, **p < *0.01*, ***p < *0.001* and ****p < *0.0001*

We next sought to assess the functionality of MuSC isolated with inhibitors by in vitro assays (Fig. 4A). After 4 days of culture in growth conditions, MuSC isolated in the presence of the inhibitors showed an increased amplification rate compared to those isolated with vehicle, this effect being more pronounced with SB + SP combination (Fig. 4B). However, only the cells purified with SB + SP formed larger colonies relative to control (Fig. 4C). Surprisingly, 48 h after seeding, we noticed that twice as many cells had proceeded to their first division when cells were isolated with inhibitors compared to vehicle (Fig. 4D). We hypothesized that restricting stress pathway activities during isolation preserved MuSC in a better state making them more inclined to adhere and divide. In line with this hypothesis, we observed that exposure to inhibitors during isolation and especially to SB (SB alone or SB + SP), led to an increased number of colonies (Fig. 4E), associated with a decreased proportion of dead cells, 48 h after plating (Fig. 4F). Lastly, after 3 days in differentiation conditions, the fusion index was similar in all conditions, indicating that exposure to the inhibitors did not affect the fusion ability of the cells (Fig. 4G, H). Interestingly, we noticed that further addition of SB + SP during the culture could potentialize the positive effect of digestion with SB + SP on MuSC amplification rate (Fig. S3A–D). Altogether, our data demonstrated that limiting P38 MAPK or JNK activation during the isolation process has a beneficial effect on MuSC function in vitro. Inhibition of P38 MAPK appeared critical for MuSC survival upon seeding, whereas combined inhibition of P38 MAPK and JNK allowed a better survival and increased amplification rate in vitro.

### MuSC exposed to P38 MAPK and JNK inhibitors during isolation exhibit increased engraftment potential in vivo

**Fig. 5:**
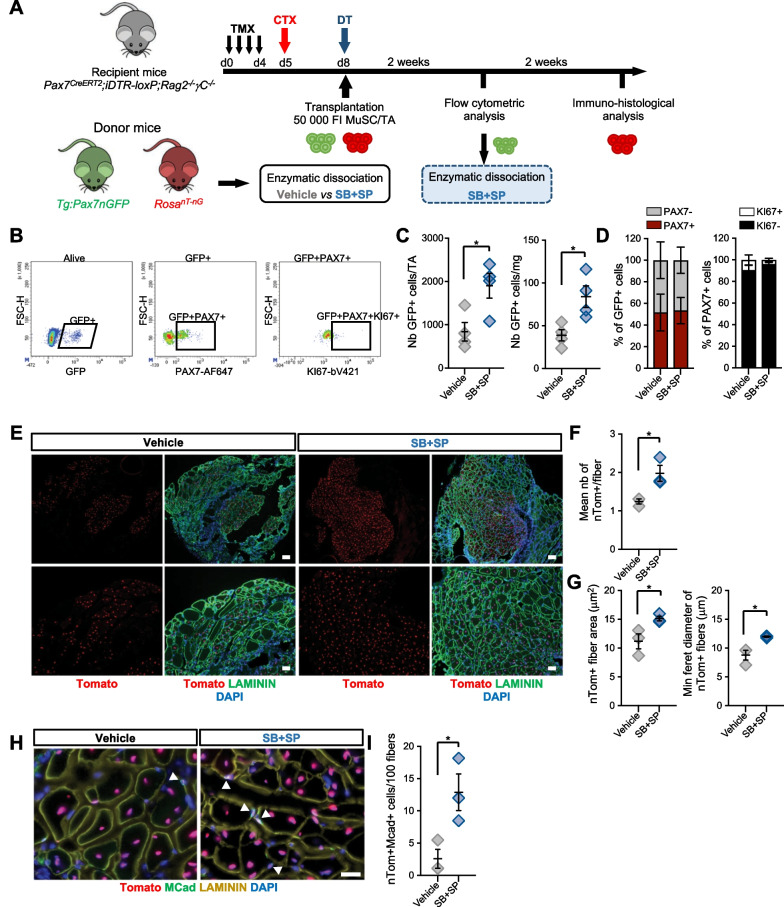
MuSC isolated with SB + SP inhibitors exhibit an increased engraftment potential. **A** Recipient mice received 4 consecutive tamoxifen (TMX) injections, followed by intramuscular injection of cardiotoxin (CTX). Diphtheria toxin (DT) was injected 3 days later (see also Methods and Fig. S4A–C). Muscles from donor mice (*Tg:Pax7nGFP* or *Rosa*^*nT−nG*^*)* were dissociated with vehicle or SB + SP, MuSC were purified by FACS and transplanted, 8 h after DT injection. Transplanted TA muscles were harvested after 2 weeks for flow cytometric analysis or 1-month for immuno-histological analysis. **B** Representative scatter plots showing the expression of PAX7 and KI67 among GFP + cells (see also Fig. S4). Quantification of **C** the total number of viable GFP + cells/TA and the number of GFP + cells/mg of TA, **D** the proportion of PAX7 + cells among the GFP + population and the proportion of KI67 + cells among the GFP + PAX7 + cells. Values are the mean ± SEM of 4 independent experiments (n = 4 mice/condition). **E** Representative images of transversal sections of TA grafted with *Rosa*^*nT−nG*^. MuSC purified with vehicle or SB + SP, 1-month post-transplantation, immunostained for Tomato and Laminin. Scale bars: 100 µm and 50 µm. Quantification of **F** the mean number of Tom + nuclei per fiber, **G** the cross-sectional area and the diameter of myofibers with Tom + nuclei. **H** Representative images of TA cross-sections immunostained for Tomato, M-Cadherin and Laminin. Arrowheads point to nTom + MCad + cells. Scale bars: 20 µm. **I** Number of nTomato + Mcadherin + cells normalized by 100 fibers. Values are the mean ± SEM of 3 independent experiments (n = 3 mice/condition). Unpaired T test and One-way ANOVA, with *p < *0.05* and **p < *0.01*

Since SB + SP treatment had the most beneficial effect on MuSC function in vitro, we next assessed its effect on their engraftment potential in vivo. MuSC were purified by FACS from two models of reporter mice, *Tg:Pax7nGFP* and *Rosa*^*nT−nG*^, in the presence of vehicle or SB + SP. Freshly isolated MuSC were transplanted to TA muscles of recipient mice previously injured by CTX injection and partially depleted of endogenous MuSC to optimize the efficacy of injected MuSC engraftment (Fig. 5A) [38, 39]. Indeed, in this recipient mouse model, Cre-driven recombination induces Diphtheria Toxin Receptor (DTR) expression specifically in PAX7 + MuSC, following tamoxifen administration. Next, intramuscular injection of Diphtheria Toxin (DT), an inhibitor of protein synthesis, induces a reproducible depletion of 75% of the endogenous MuSC (Fig. S4A–C). Two weeks post-transplantation, the number of engrafted mononucleated GFP + cells was determined by flow cytometry (Fig. 5B and Fig. S4D–G). We observed that the total number of GFP + cells/TA as well as the number of GFP + cells normalized per mg of muscle, was significantly increased when MuSC had been processed with SB + SP during the whole isolation process (Fig. 5C). The mean proportion of PAX7 + cells among the GFP + population was equivalent between vehicle and SB + SP (Fig. 5D). Moreover, regardless of the treatment more than 90% of the GFP + PAX7 + cells were KI67- and thus presumably in a quiescent state (Fig. 5D). Based on these observations and in vitro data, we concluded that the increased number of GFP + cells in SB + SP condition, was mainly due to an improved survival and/or amplification of the transplanted MuSC rather than an increased self-renewal capacity. We next analyzed the engraftment efficacy 1-month post-transplantation of *Rosa*^*nT−nG*^ MuSC isolated with SB + SP or its vehicle, by immunofluorescence (Figs. 5E and S4H, I). We noticed that the mean number of Tomato + nuclei (nTom +) per myofiber and the size of these myofibers were significantly increased in SB + SP compared to vehicle condition (Fig. 5F, G). Consistent with our data at 2 weeks, we observed a higher number of sub-laminal nTom + MCadherin + cells in SB + SP condition than in vehicle (Figs. 5H, I and S4J). Overall, these experiments demonstrate that combined inhibition of P38 MAPK and JNK during isolation preserves the regenerative potential of MuSC.

### An optimized version of muscle tissue dissociation protocol enabling high purification yield of human MuSC while preserving their stemness

**Fig. 6:**
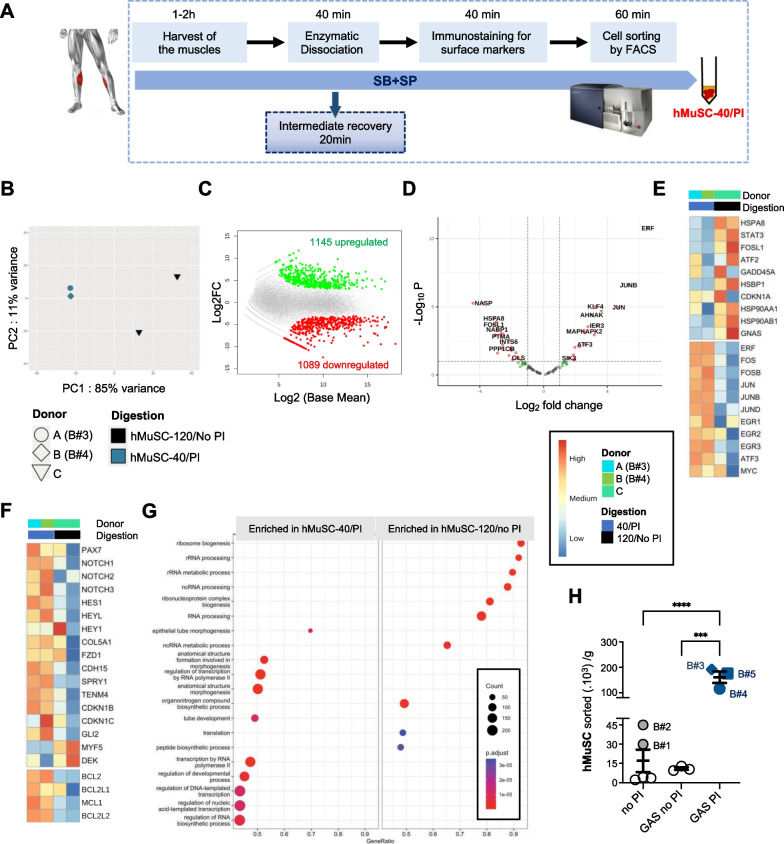
Optimized muscle dissociation protocol enables marked improvement of purification yield of hMuSC with preserved stemness. **A** Human muscle biopsies were processed with SB + SP from the harvest to the FACS sorting. hMuSC (Lin-CD34-CD29 + CD56 + ITGA7 + , see Fig . S5A–D) purified with this protocol are referred to as hMuSC-40/PI (40 min dissociation, with Pharmacological Inhibitors). **B**–**G** Comparative analysis of the transcriptome of freshly isolated hMuSC-40/PI to that of hMuSC isolated without PI previously published (hMuSC-120/no PI) [25], analyzed by RNAseq. **B** PCA plot of gene expression data. **C** MA plot of differentially expressed genes (DEG). **D** Volcano plot of 100 stress core genes differentially expressed. Heatmap clustering profiles of **E** stress core target genes, **F** quiescence and anti-apoptotic genes, based on log_2_ transformed counts. **G** Top biological pathways enriched hMuSC-40/PI *versus* hMuSC-120/no PI, based on differentially expressed genes and ranked by *p* value. **H** Comparison of hMuSC purification yield, when muscles were digested without inhibitors (no PI) or with SB + SP (PI). Grey and blue filled symbols represent our data (Biopsies 1–5, B#1 to B#5), open black circles correspond to data extracted from the literature. GAS: Gastrocnemius. See also Table S1. Values are the mean ± SEM of 3–5 independent biopsies, One-way ANOVA and Tukey’s multiple comparisons test, ***p < *0.001* and ****p < *0.0001*

We next sought to determine whether our findings were relevant to human MuSC (hMuSC). To alleviate the detrimental impact of long-term enzymatic dissociation, we optimized the standard protocol used in the field. We restricted the dissociation time to 40 min, performed an intermediate recovery of mononucleated cells at 20 min, and added SB + SP during the whole isolation process. hMuSC were sorted by FACS using CD29, CD56, and ITGA7 as selection markers (Fig. 6A and Fig. S5A–D). Given the small size and the rarity of human biopsies, to assess the effect of this optimized purification protocol, we compared the transcriptome of hMuSC purified following this protocol (referred to as hMuSC-40/PI) to that of hMuSC isolated after 120 min of dissociation without inhibitors previously published (hMuSC-120/no PI) [25], by RNA sequencing. Principal component analysis (PCA) plot of gene expression revealed distinct transcriptomic profiles between the 2 groups (Fig. 6B), with more than 2000 differentially expressed genes (DEG) (Fig. 6C). Interestingly, hMuSC-40/PI samples coming from 2 distinct donors clustered together, whereas the 2 hMuSC-120/no PI samples corresponding to a biological replicate of the same donor were more distant, suggesting that our isolation strategy could reduce the variability between samples. By comparing the expression profiles of the stress core genes [17], we identified two gene sets (Fig. 6D, E). Genes which expression was reduced in hMuSC-40/PI, including known target genes of P38 MAPK and JNK (*HSPB1, HSP90A*, *GADD45*, *ATF2*), as well as *FOSL1* and *CDKN1a* that were also down-regulated in mMuSC isolated with SB + SP (Fig. 6E). In contrast, *EGR* and AP-1 gene expression was globally higher in hMuSC-40/PI, likely due to the different dissociation times (Fig. 6E). Indeed, the expression of *FOS* and *JUN* genes is peaking at 40 min of dissociation, whereas it is declining at 120 min [17, 32]. Expression of genes involved in MuSC stemness and quiescent state was systematically higher in hMuSC-40/PI including, *PAX7, CDH15, SPRY1, COL5a, TENM4,* Notch receptors and downstream target genes (*HES1, HEY1, HEYL*) [45]. hMuSC-40/PI exhibited increased expression of anti-apoptotic genes (*BCL2, BCL2L1, BCL2L2* and *MCL1*). Transcripts of *MYF5, DEK* and inflammation-related genes were up-regulated in hMuSC-120/no PI, as previously described in an early activated population of hMuSC [46] (Figs. 6F and S5E, F). Lastly, pathways associated with protein translation and ribosomes were enriched in hMuSC-120/no PI, whereas pathways related to gene transcription were enriched in hMuSC-40/PI (Fig. 6G). Overall, this comparative study indicates that our protocol favors the purification of hMuSC with preserved expression of stemness markers, better survival and in a less activated state than standard protocols, in agreement with our data on mMuSC.

To assess whether these beneficial effects could translate into a better purification yield of hMuSC, we took advantage of previously published data [13, 14, 47] (Table S1). We noticed a marked improvement of hMuSC purification yield when human biopsies were processed following our protocol, compared to data extracted from the literature or to the data we previously obtained with standard dissociation protocol (Fig. 6H). Therefore, our purification strategy could greatly ameliorate hMuSC purification yield (up to 14-fold).

## Discussion

The quiescent state of adult MuSC relies on complex cellular and molecular interactions that guarantee their stemness and therefore their regenerative potential. In recent years, the transcriptome of quiescent MuSC has been thoroughly revisited, notably through a better understanding of the molecular signaling events triggering the early activation of these cells upon muscle tissue dissociation and acute injury [16–18, 20, 32]. Our laboratory has identified a non-specific stress core response, common to most digested tissues, triggered by the massive activation of stress signaling pathways, including P38 MAPK, JNK and ERK [17]. Since enzymatic dissociation of muscle tissue is necessary for the purification of these cells, a better understanding of the impact of these stress pathways on the stemness of MuSC could provide new avenues for their use in clinic. However, delineating the role of P38 MAPK and JNK can be complex, as these 2 pathways are induced by the same stress signals and interact with each other in different manners depending on the circumstances and cell types. In this study, we sought to uncouple P38 MAPK and JNK activities in order to decipher their respective role during dissociation-induced MuSC activation. We showed that a two-step decline of PAX7 protein occurs during enzymatic dissociation of muscle. P38 MAPK and JNK were only involved in the second phase, which was slower and heterogeneous across the MuSC population, in agreement with the heterogeneity of the stress index previously reported [18]. The mechanisms underlying this rapid loss of PAX7 protein remains unknown. Several mechanisms involved in PAX7 post-translational regulation have been described [48–51]. For instance, NEDD4, a ubiquitin-ligase, was shown to negatively regulate PAX7 protein [48]. Since NEDD4 and Rac1 were shown to cooperate in cell–cell contacts, it might be of interest to investigate whether this partnership could be involved in PAX7 protein regulation upon cytoplasmic projection retraction of MuSC [52]. Accordingly, addition of pharmacological inhibitors of these two pathways alone or in combination, maintained detectable levels of PAX7 protein in a higher number of MuSC during their isolation process.

We observed that our treatment, had moderate impact on the induction of immediate-early genes. Expression of *Fos* gene, considered as the first gene induced by muscle dissociation, was unaffected by SB and/or SP treatment, in agreement with previous study [16, 21]. We showed that *Egr3* gene was partly induced by JNK, whereas P38 MAPK contributed to *Junb* induction, and both JNK and P38 MAPK were involved in *Fosb* and *Fosl1* gene induction. Despite, modest reduction in *Egr* and AP-1 family member expression, we showed that combined inhibition of p38 MAPK and JNK, could restrained early activation of MuSC, evidenced by a significant reduction of *Myod1* and *Cdkn1a* transcripts and increased level of *Pax7* and *CalcR* transcripts in murine MuSC. Interestingly, upon proliferation-to-differentiation transition, P38 MAPK was found to be recruited to *Pax7* and *Myod1* promoters where its acts as a negative or a positive transcription regulator, respectively [28]. P38 MAPK can repress *Pax7* gene transcription through the phosphorylation the epigenetic silencer EZH2 by [53]. In addition, activated JNK was able to prevent CDK1-mediated phosphorylation and subsequent degradation of EZH2 by SMURF2 in erythroblasts [54]. We therefore hypothesize that the increased effect of combined SB + SP treatment on *Pax7* transcript level could result from a more pronounced inhibition of EZH2. Additional experiments will be needed to determine whether *Pax7* transcript level is regulated through EZH2 pathway or another mechanism during muscle dissociation. Indeed, previous study suggested that RNA degradation rather than gene transcription may be predominantly involved in the transcriptome changes during dissociation-induced early activation of MuSC [55]. In agreement, P38 MAPK and JNK were both involved in the stability of mRNA harboring adenylate- and uridylate (AU)-rich elements (ARE) in their 3’-UTR sequence, such as some members of AP-1 family, *Cdkn1a, Bcl2* and *EGFR* [56]. Such a regulation of mRNA is thus likely to occur during early activation of MuSC through P38 MAPK and JNK or other kinases induced during this process.

Importantly, we demonstrated that JNK activation induces a decrease of CD34 surface protein, which is essential for murine MuSC functionality and quiescent state [43]. We anticipate that this effect could rely on a post-translational regulation since a putative phosphorylation site by JNK was identified in the intracellular domain of CD34 [44]. However, this finding is not relevant for human MuSC that are considered to be mainly negative for CD34 [13].

By in vitro assays, we showed that inhibition of P38 MAPK and JNK during the whole isolation process of mMuSC had a positive impact on their survival and amplification rate. In the same manner, mMuSC isolated with SB + SP exhibited increased survival and engraftment potential upon transplantation in vivo. Our findings provide new insights on the impact of dissociation-induced stress signaling pathways activation, by clarifying the respective impact of P38 MAPK and JNK during this process. More importantly, we have proven that slowing down the transition from quiescence to the activated state of MuSC through pharmacological inhibition of these pathways was beneficial and did not affect their regenerative potential. Accordingly, it would be of particular interest to assess whether combined inhibition of P38 MAPK, JNK, ERK and Rho GTPases could have a synergic effect on the preservation of MuSC regenerative potential during their purification.

Our comparative approach on hMuSC has limitations since we could not firmly distinguish between the effect of dissociation time and SB + SP treatment. However, taking advantage of previously published RNAseq resources, we were able to provide evidence that this method of purification may also be suitable to ameliorate the isolation of hMuSC with preserved expression of stemness markers and better survival.

## Conclusion

Overall, our study highlights the extent to which the MuSC purification step is crucial to preserve their functionality. We therefore propose a revised version of the dissociation protocol commonly used in the field, enabling to improve MuSC purification yield and preserve their regenerative potential. This protocol complies with the 3R rules, since it will contribute to reduce the number of animals used for fundamental research in the muscle field. We anticipate that our findings will also be a valuable asset for isolating more potent hMuSC for clinical applications such as cell-based therapies and regenerative medicine.

### Supplementary Information

Supplementary Material 1.

## Data Availability

The accession numbers for RNA-seq raw and processed files can be downloaded from the Gene Expression Omnibus website (https://www.ncbi.nlm.nih.gov/geo/) under the accession number GEO: GSE268475.

## Abbreviations

3R: Replace, reduce, refine
CTX: Cardiotoxin
DT: Diphtheria toxin
EGR: Early growth response
ERK: Extracellular regulated kinases
EZH2: Enhancer of zeste homolog 2
GAS: Gastrocnemius muscle
GFP: Green fluorescent protein
hMuSC: Human muscle stem cells
IM: Intramuscular
IP: Intraperitoneal
JNK: C-Jun N-terminal kinases
MAPK: Mitogen activated protein kinase
MHC: Myosin heavy chain
mMuSC: Murine muscle stem cells
mRNA: Messenger RNA
MuSC: Muscle stem cells
MYOD: Myogenic differentiation 1
MYOG: Myogenin
NK: Natural killer
PAX7: Paired-Homeobox transcription factor 7
PI: Pharmacological inhibitors
RNA: Ribonucleic acid
RNA seq: RNA sequencing
RT-qPCR: Real time quantitative polymerase chain reaction
SC: Satellite cells
SB: SB202190
SP: SP600125
TMX: Tamoxifen
TOM: Tomato
