# Supplementary material for: Dual inhibition of P38 MAPK and JNK pathways preserves stemness markers and alleviates premature activation of muscle stem cells during isolation
Source: Stem Cell Res Ther. 2024 Jun 21;15:179. doi: 10.1186/s13287-024-03795-0 (PMC11191274; doi:10.1186/s13287-024-03795-0)
Supplement: Supplementary file 1 — Supplementary Material 1. [file 13287_2024_3795_MOESM1_ESM.pdf]

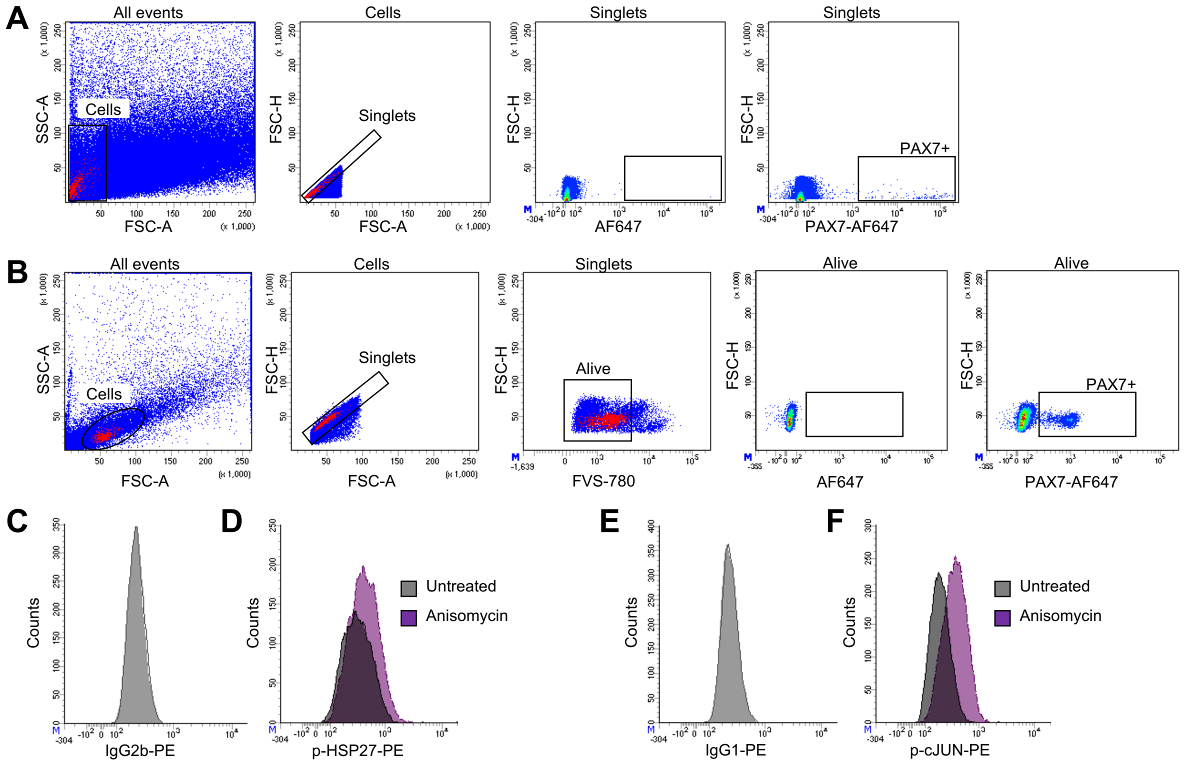

Figure S1 related to Figure 1

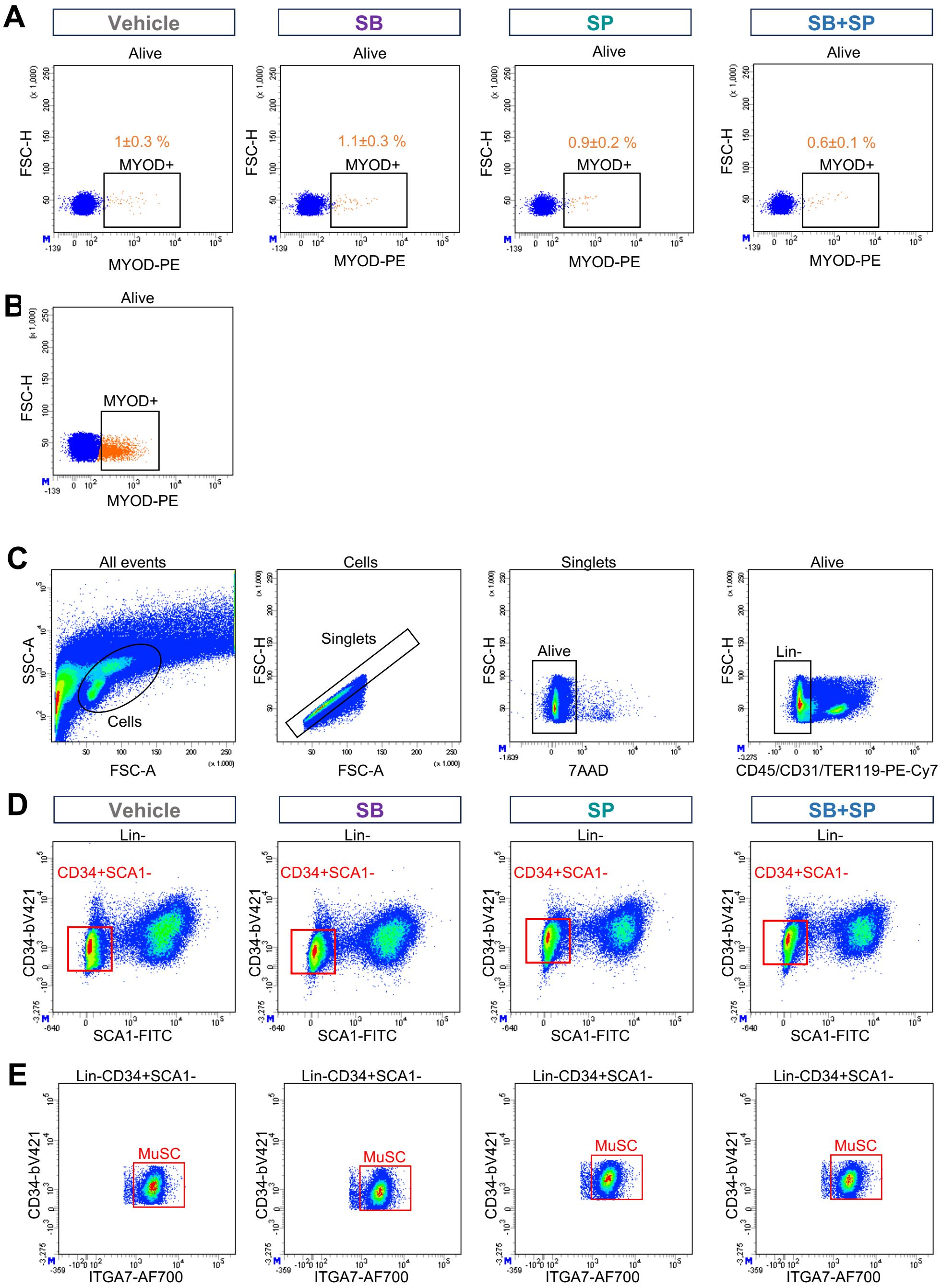

Figure S2 related to Figure 3

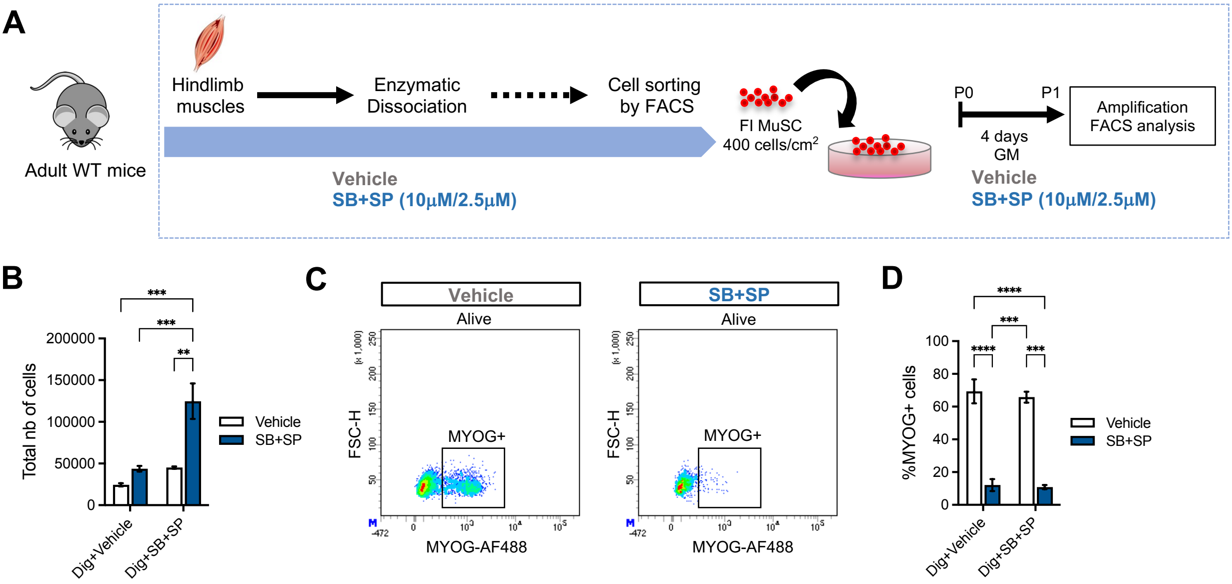

Figure S3 related to Figure 4

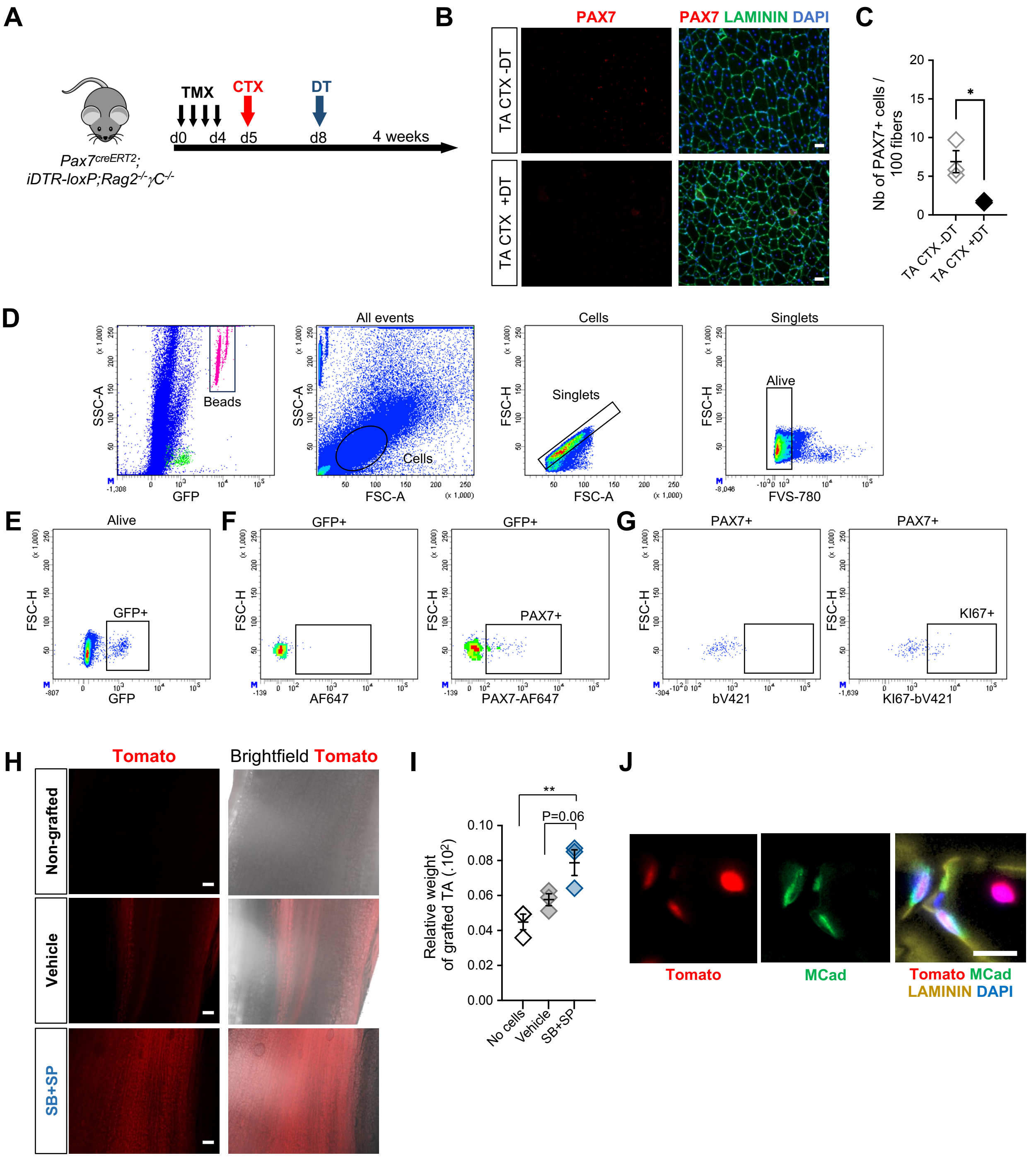

Figure S4 related to Figure 5

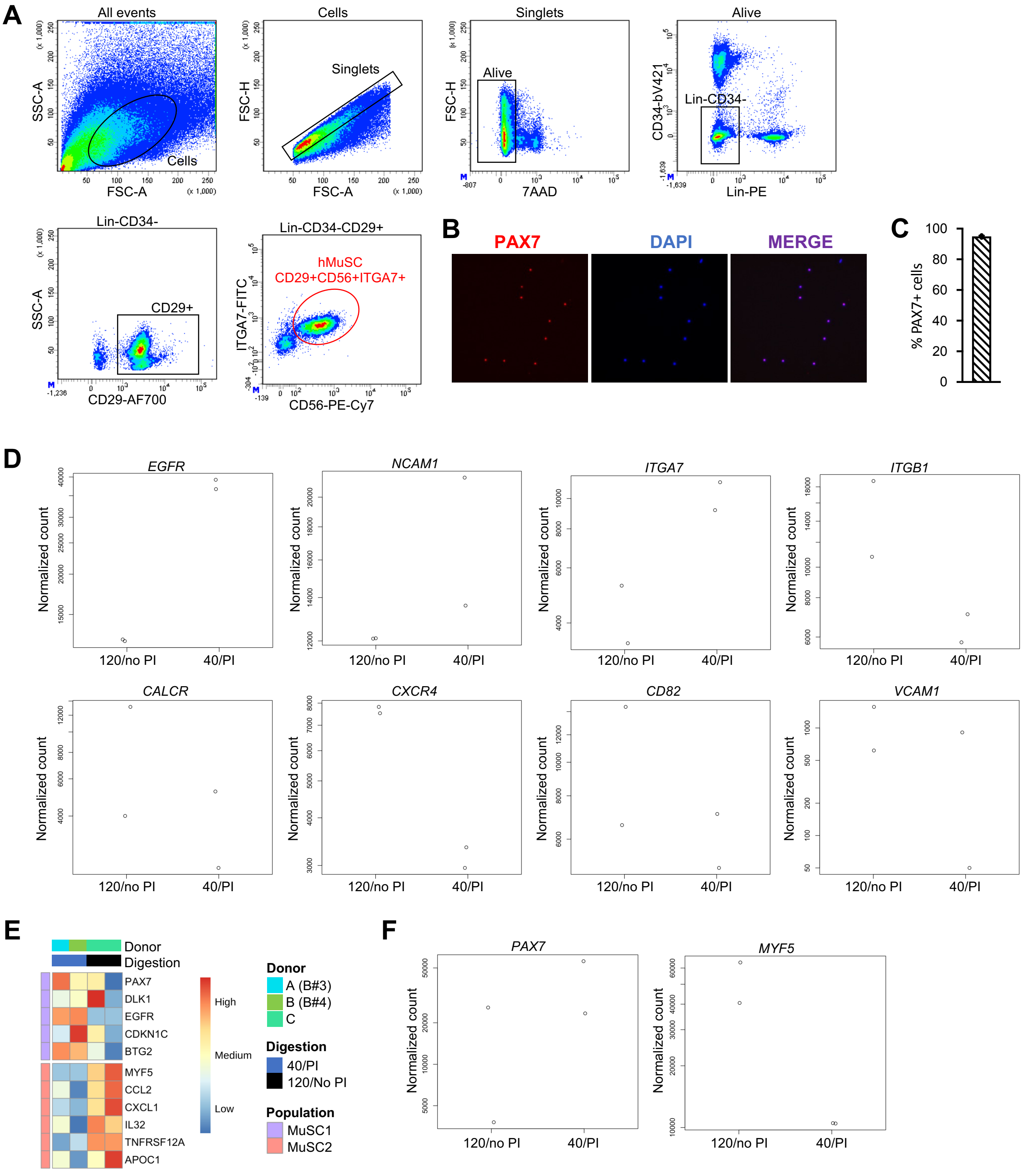

Figure S5 related to Figure 6

Supplemental figure legends

Figure S1 related to Figure 1: Analysis of PAX7 and phosphorylated forms of HSP27 and cJUN, by flow cytometry. (**A**) Representative scatter plots showing the gating strategy to analyze PAX7 expression in mononucleated cells from TA fixed immediately after harvest (T0). Debris and doublets were excluded, and PAX7 positivity was determined based on FMO control. (**B**) Representative scatter plots showing the gating strategy to analyze PAX7 expression in mononucleated cells from TA dissociated for 40 minutes before fixation. Debris, doublets and dead cells were excluded from the analysis, and PAX7 positivity was determined based on FMO control. (**C-F**) Validation of anti-phospho-HSP27 and anti-phospho-cJUN coupled antibody specificities. C2C12 myogenic cells were treated for 1 hour with anisomycin 5 µM or vehicle, fixed and permeabilized, and then immunostained with coupled isotypic controls (**C**, **E**) or with coupled antibodies against phospho-HSP27 (**D**) or phospho-cJUN (**F**). Debris, doublets and dead cells were excluded from the analysis.

Figure S2 related to Figure 3. Lowering P38 and JNK pathway activities during dissociation and sorting steps preserves stemness marker expression and limits early activation in MuSC. (**A**) Representative scatter plots showing MYOD expression in mononucleated cells from muscles dissociated with Vehicle, SB, SP or SB+SP, analyzed by flow cytometry as previously described (1). (**B**) MYOD expression in mononucleated cells from postnatal mouse muscles, was used as positive control. Debris, doublets and dead cells were excluded from the analysis. (**C**) Strategy to purify murine MuSC by FACS. 7AAD was used as live dye. Debris, doublets, dead cells and CD45^{+}Ter119^{+}CD31^{+} (Lin+) were excluded from the analysis and ITGA7^{+} MuSC were gated from the CD34^{+}SCA1^{-} fraction. (**D, E**) Representative density scatterplots

showing the expression of CD34 among the Lin-SCA1- fraction (**D**) and of ITGA7 among the Lin-CD34+SCA1- populations (**E**), in mononucleated cells from muscles dissociated with Vehicle, SB, SP or SB+SP.

Figure S3 related to Figure 4. Beneficial effect of SB+SP digestion on MuSC amplification is extended by the addition of the inhibitors in the culture medium. (**A**) Experimental procedure. MuSC were purified with vehicle or both SB+SP inhibitors and plated at low density in Growth Medium (GM) complemented with SB+SP inhibitors or Vehicle for 4 days. Medium were changed at 48h. (**B**) Graph showing the total number of cells after 4 days of amplification. (**C**) Representative density scatterplots showing the proportion of MYOG+ cells in MuSC amplified with SB+SP or Vehicle, in GM for 4 days. (**D**) Graph showing the percentage of MYOG+ cells. Values are the mean ± SEM of minimum 3 independent experiments (n=3-4 mice/group). Two-way ANOVA analysis, with ***p<0.01*, ****p<0.001* and *****p<0.0001*.

Figure S4 related to Figure 5: MuSC isolated with SB+SP inhibitors exhibit an increased engraftment potential. (**A**) Experimental procedure to assess the efficacy of endogenous MuSC depletion in *Pax7*^{creERT2}*;iDTR-loxP;Rag2*$^{-/-}$*;γC*$^{-/-}$ recipient mice. Tamoxifen was injected intraperitoneally for 4 consecutive days to induce Cre-driven recombination, and expression of the DTR by endogenous PAX7+ MuSC. The next day, Cardiotoxin (CTX) was injected in both TA muscles, and 3 days later Diphtheria toxin (DT) was injected solely in one TA (TA CTX +DT). (**B**) Representative images of TA muscle sections, immunostained for PAX7 and LAMININ, 1-month post-injury. Nuclei were stained with DAPI. Scale bar: 200 µM. (**C**) Total number of PAX7+ cells normalized per 100 fibers. Injection of DT resulted in a 75% depletion of endogenous

PAX7+ MuSC. (**D-G**) Representative dot and density plots showing the gating strategy used for quantitative analysis of engrafted GFP+ cells on single TA muscle, by flow cytometry. (**D**) Acquisitions were performed on 20 000 beads, using Trucount tubes. Debris, doublets and dead cells were excluded. Representative density plots showing, the GFP+ cells (**E**), FMO and PAX7 staining on GFP+cells (**F**), FMO and KI67 staining on PAX7+ cells (**G**). (**H**) Images of whole TA muscles 1-month after transplantation with *Rosa*$^{nT\text{-}nG}$ MuSC purified with vehicle or SB+SP. Scale bars: 100μm. (**I**) Relative weight of transplanted TA muscles upon harvest. (**J**) Images showing the presence of sub-laminal nTom+Mcadherin+ MuSC, 1-month post-transplantation. Scale bar: 10μm.

Figure S5 related to Figure 6: An optimized version of muscle dissociation protocol enables marked improvement of purification yield of human MuSC with preserved stemness. (**A**) Strategy to purify human MuSC by FACS. 7AAD was used as live dye. Debris, doublets, dead cells, $CD45^{+}CD31^{+}CD11b^{+}CD235a^{+-}$ (Lin+) and $CD34^{+}$ cells were excluded, $CD56^{+}ITGA7^{+}$ hMuSC were gated from the CD29+ fraction. (**B**) hMuSC immunostainined for PAX7 24h after sorting. (**C**) Proportion of PAX7+ cells 24h after sorting. (**D**) Normalized counts obtained for known surface markers of hMuSC (2–6), by RNAseq analysis on hMuSC-120/no PI (120/no PI) *versus* hMuSC-40/PI (40/PI). (**E**) Heatmap clustering profiles of MuSC1 and MuSC2 associated genes based on $\log_2$ transformed counts to identify consistent changes in expression profiles between hMuSC-40/PI and hMuSC-120/no PI. MuSC1 (quiescent) and MuSC2 (activated) populations were previously identified by scRNAseq on human muscle biopsies (7). (**F**) Normalized counts obtained for *PAX7* and *MYF5* in hMuSC-120/no PI *versus* hMuSC-40/PI.

Supplemental tables

Table S1 relative to Figure 6: Characteristics of human biopsies

Biopsy ID	Age	Sex	Muscle	Surface markers	MuSC/g	Group
Biopsy 3 (B#3)	25	F	Gastrocnemius	$CD56^{+}/CD29^{+}/ITGA7^{+}$	167 000/216 000	GAS PI
Biopsy 4 (B#4)	41	F	Gastrocnemius	$CD56^{+}/CD29^{+}/ITGA7^{+}$	92 000/140 000	GAS PI
Biopsy 5 (B#5)	25	M	Gastrocnemius	$CD56^{+}/CD29^{+}/ITGA7^{+}$	175714	GAS PI
Garcia et al., 2018	20	M	Gastrocnemius	$CXCR4^{+}/CD29^{+}/CD56^{+}$	12 945	GAS no PI
Garcia et al., 2018	43	M	Gastrocnemius	$CXCR4^{+}/CD29^{+}/CD56^{+}$	10 453	GAS no PI
Garcia et al., 2018	59	M	Gastrocnemius	$CXCR4^{+}/CD29^{+}/CD56^{+}$	9 525	GAS no PI
Biopsy 1 (B#1)	45	M	Hip muscle	$CD56^{+}/CD29^{+}/ITGA7^{+}$	30 000	No PI
Biopsy 2 (B#2)	53	M	Hip muscle	$CD56^{+}/CD29^{+}/ITGA7^{+}$	45 000	No PI
Gheller et al., 2021	NS	NS	NS	$CD56^{+}/CD29^{+}$	4 267	No PI
Gheller et al., 2021	NS	NS	NS	$CD56^{+}/CD29^{+}$	2 306	No PI
Castiglioni et al., 2014	NS	NS	NS	$CD56^{int}ITGA7^{hi}$	3 600	No PI

GAS: Gastrocnemius – PI: Pharmacological Inhibitors - NS: Not Specified

Table S2. List of antibodies used for flow cytometry analysis

Antibody	Reference	Brand
Mouse anti-PAX3/7-AF647 (B-5)	#sc-365843 AF647	Santa Cruz
Mouse anti-phospho-HSP27 (B-3)	#sc-166693 PE	Santa Cruz
Mouse anti-phospho-c-JUN (KM-1)	#sc-822 PE	Santa Cruz
Mouse anti-MYOD-PE	#554130	BD Biosciences
Mouse anti-MYOG-AF488 (5FD)	#sc-52903	Santa Cruz
Mouse IgG2a-AF647	#sc-24637 AF647	Santa Cruz
Mouse IgG2b-PE	#sc-2868 PE	Santa Cruz
Mouse IgG1-PE	sc-2866 PE	Santa Cruz
Mouse IgG1-PE	#550617	BD Biosciences
Mouse IgG1-AF488	#sc-3890	Santa Cruz

Table S3. List of coupled antibodies used for mouse MuSC sorting

Antibody	Reference	Brand
Rat anti-mouse CD45-PECy7	#552848	BD Biosciences
Rat anti-mouse TER-119-PECy7	#557853	BD Biosciences
Rat anti-mouse CD31-PE-Cy7	#561410	BD Biosciences
Rat anti-mouse CD34-bV421	#562608	BD Biosciences
Rat anti-mouse SCA-1-FITC	#553335	BD Biosciences
Mouse anti-mouse ITGA7-AF700	#FAB3518N	R&D Systems

Table S4. List of coupled antibodies used for human MuSC sorting

Antibody	Reference	Brand
Mouse anti-human CD45-PE	#555482	BD Biosciences
Mouse anti-human CD235a-PE	#555570	BD Biosciences
Mouse anti-human CD11b-PE	#555388	BD Biosciences
Mouse anti-human CD31-PE	#555466	BD Biosciences
Mouse anti-human CD34-bv421	#562577	BD Biosciences
Mouse anti-human CD56-PE-Cy7	#557747	BD Biosciences
Mouse anti-human CD29-AF700	#303019	Biolegend
Mouse anti-mouse ITGA7-FITC	#LS-C179570	LSBio

Table S5. List of antibodies used for immunostaining on sections

Primary Antibodies	Reference	Brand	Final Dilution
mouse IgG1 anti-RFP	#M155-3	MBL Life Science	1:100
rabbit anti-Laminin	#L9393	Sigma	1:200
goat anti-TdTomato	#ABIN6254170	Antibody Online	1:100
mouse IgG1 anti-M-cadherin	#sc-81471	Santa Cruz	1:100
mouse IgG2b anti-Myosin Heavy Chain (MF20)	MAB4470	R&D Systems	1:100
mouse IgG1 anti-PAX7	#sc-81648	Santa Cruz	1:100
**Secondary antibodies**	**Reference**	**Brand**	**Final Dilution**
Goat anti-Mouse IgG1-Cy3 (GaM1-Cy3)	#115-165-205	Jackson Immunoresearch	1:1000
Goat anti-Rabbit-AF488	#111-545-144	Jackson Immunoresearch	1:1000
Donkey anti-Goat-Cy3	#705-165-147	Jackson Immunoresearch	1:1000
Goat anti-Mouse IgG1-AF488 (GaM1-AF488)	#115-545-205	Jackson Immunoresearch	1:1000
Goat anti-Mouse IgG2b -Cy3	#115-165-207	Jackson Immunoresearch	1:1000
Goat anti-mouse immunoglobulin G (IgG) Fab fragment	#115-007-003	Jackson Immunoresearch	1:100

Table S6. List of the primers used for RT-qPCR

Gene ID	Forward Primer 5’-3’	Reverse Primer 3’-5’
Fos	GCTGACAGATACACTCCAAGCG	GCAGATTGGCAATCTCAGTCTGC
Fosb	AGCTAAGTGCAGGAACCGTC	ACTCCAGCTCTGCCTTTTCC
Fosl1	ATGTACCGAGACTACGGGGAA	CTGCTGCTGTCGATGCTTG
Junb	AGGCAGCTACTTTTCGGGTC	TTGCTGTTGGGGACGATCAA
cjun	GGAACAGGTGGCACAGCTTA	GCTGCGTTAGCATGAGTTGG
Hspb1	ATCCCCTGAGGGCACACTTA	GGAATGGTGATCTCCGCTGAC
Cdkn1a	TGGAGTCAGGCGCAGATCCAC	CGCCATGAGCGCATCGCAATC
MyoD	GGCTACGACACCGCCTACTA	GAGATGCGCTCCACTATGCT
Pax7	AGGCCTTCGAGAGGACCCAC	CTGAACCAGACCTGGACGCG
CalcR	TCATCATCCACCTGGTTGAG	GCTCGTCGGTAAACACAGC

Spry1	GAGGCCGAGGATTTCAGATGCA	CTGAATCACCACTAGCGAAGTGT
Cd34	AGGCTGATGCTGGTGCTAG	AGTCTTTCGGGAATAGCTCTG
Egr1	GAGCACCTGACCACAGAGTC	CGAGTCGTTTGGCTGGGATA
Egr2	TGACCAGATGAACGGAGTGG	GCGAAGCTACTCGGATACGG
Egr3	CCGGTGACCATGAGCAGTTT	TAATGGGCTACCGAGTCGCT
Po	CTCCAAGCAGATGCAGCAGA	ATAGCCTTGCGCATCATGGT

Supplemental methods

Bioinformatic analysis of RNAseq data. cDNA libraries were generated with 1 ng of total RNA using the Illumina Stranded total RNA prep Ligation kit. FASTQ files were aligned to the human reference genome (Ensembl Homo Sapiens GRC38.110 assembly) using the Hisat2 aligner software (Hisat2.2.1) with default parameters (8), and exported into BAM format (9). 20 to 30 million reads per sample were obtained, with more than 85% mapping efficiency. FeatureCounts was used to obtain the number of aligned reads per gene(10). To calculate differential gene expression, gene read count tables generated by FeatureCounts were used as input for the R package DESeq2 (11). For the comparative analysis using RNA-Seq data from hMuSC (4), the same bioinformatic pipeline was applied. Sex-dependent genes were discarded from the samples before processing to DESeq2 analysis in R. Gene set enrichment analysis was performed using ClusterProfiler package in R (12).

Supplemental references

1. Gattazzo F, Laurent B, Relaix F, Rouard H, Didier N. Distinct Phases of Postnatal Skeletal Muscle Growth Govern the Progressive Establishment of Muscle Stem Cell Quiescence. Stem Cell Rep. 8 sept 2020;15(3):597-611.
2. Castiglioni A, Hettmer S, Lynes MD, Rao TN, Tchessalova D, Sinha I, et al. Isolation of progenitors that exhibit myogenic/osteogenic bipotency in vitro by fluorescence-activated cell sorting from human fetal muscle. Stem Cell Rep. 14 janv 2014;2(1):92-106.

3. Gheller BJ, Blum J, Soueid-Baumgarten S, Bender E, Cosgrove BD, Thalacker-Mercer A. Isolation, Culture, Characterization, and Differentiation of Human Muscle Progenitor Cells from the Skeletal Muscle Biopsy Procedure. J Vis Exp. 23 août 2019;(150):59580.
4. Charville GW, Cheung TH, Yoo B, Santos PJ, Lee GK, Shrager JB, et al. Ex Vivo Expansion and In Vivo Self-Renewal of Human Muscle Stem Cells. Stem Cell Rep. 13 oct 2015;5(4):621-32.
5. Garcia SM, Tamaki S, Lee S, Wong A, Jose A, Dreux J, et al. High-Yield Purification, Preservation, and Serial Transplantation of Human Satellite Cells. Stem Cell Rep. 13 mars 2018;10(3):1160-74.
6. Alexander MS, Rozkalne A, Colletta A, Spinazzola JM, Johnson S, Rahimov F, et al. CD82 Is a Marker for Prospective Isolation of Human Muscle Satellite Cells and Is Linked to Muscular Dystrophies. Cell Stem Cell. déc 2016;19(6):800-7.
7. De Micheli AJ, Spector JA, Elemento O, Cosgrove BD. A reference single-cell transcriptomic atlas of human skeletal muscle tissue reveals bifurcated muscle stem cell populations. Skelet Muscle. déc 2020;10(1):19.
8. Kim D, Paggi JM, Park C, Bennett C, Salzberg SL. Graph-based genome alignment and genotyping with HISAT2 and HISAT-genotype. Nat Biotechnol. août 2019;37(8):907-15.
9. Li H, Handsaker B, Wysoker A, Fennell T, Ruan J, Homer N, et al. The Sequence Alignment/Map format and SAMtools. Bioinforma Oxf Engl. 15 août 2009;25(16):2078-9.
10. Liao Y, Smyth GK, Shi W. featureCounts: an efficient general purpose program for assigning sequence reads to genomic features. Bioinformatics. 1 avr 2014;30(7):923-30.
11. Love MI, Huber W, Anders S. Moderated estimation of fold change and dispersion for RNA-seq data with DESeq2. Genome Biol. 5 déc 2014;15(12):550.
12. Yu G, Wang LG, Han Y, He QY. clusterProfiler: an R package for comparing biological themes among gene clusters. Omics J Integr Biol. mai 2012;16(5):284-7.
